# Pruritic Crusted Scabies in an Immunocompetent Infant

**DOI:** 10.1155/2019/9542857

**Published:** 2019-10-20

**Authors:** Alexander K. C. Leung, Kin Fon Leong, Joseph M. Lam

**Affiliations:** ^1^University of Calgary and Alberta Children's Hospital, Calgary, Alberta, T2M 0H5, Canada; ^2^Pediatric Institute, Kuala Lumpur General Hospital, Kuala Lumpur, Malaysia; ^3^Department of Dermatology and Skin Sciences, University of British Columbia and BC Children's Hospital, Vancouver, Canada

## Abstract

Crusted scabies (also known as Norwegian scabies) is a highly contagious variant of scabies characterized by profuse proliferation of mites in the skin and widespread, crusted, hyperkeratotic papules, plaques, and nodules. Typically, pruritus is minimal or absent. The condition usually occurs in immunocompromised individuals. Occurrence in healthy infants has rarely been reported. We report an 11-month-old healthy Malay boy who presented with crusted scabies.

## 1. Introduction

Human scabies is a skin infestation caused by an obligate human parasite mite *Sarcoptes scabiei var hominis* that results in an intensely pruritic eruption with a characteristic distribution pattern [1]. Crusted scabies (also known as Norwegian scabies and hyperkeratotic scabies) is a highly contagious and severe form of scabies characterized by profuse proliferation of mites in the skin and widespread, crusted, hyperkeratotic papules, plaques, and nodules [2–5]. Typically, pruritus is minimal or absent [2, 6]. Crusted scabies usually occurs in immunocompromised individuals [2, 5]. Occurrence in healthy infants has rarely been reported. We report an 11-month-old healthy Malay boy who presented with crusted scabies; the lesions of which were intensively pruritic.

## 2. Case Report

**Figure 1:**
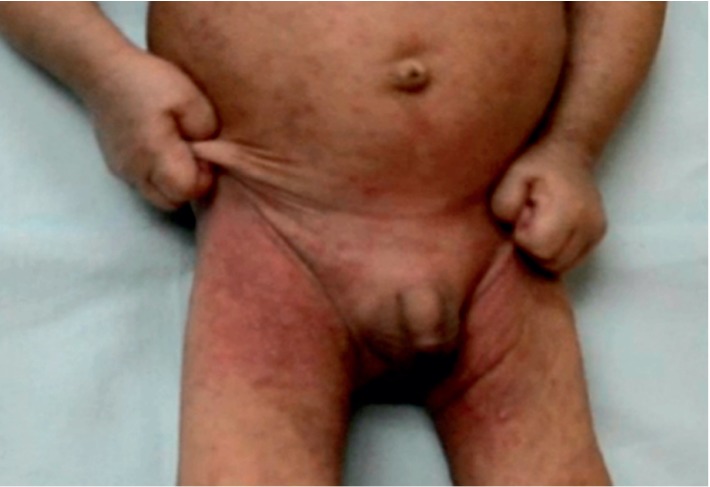
An 11-month-old infant with an intensely pruritic rash was noted to pinch his skin constantly.

**Figure 2:**
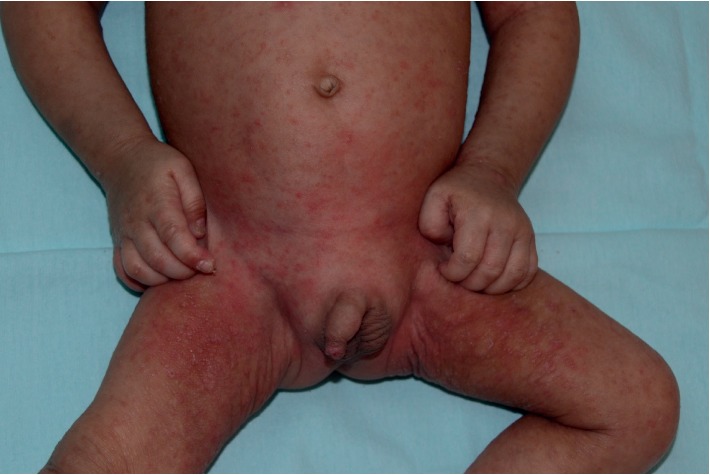
An 11-month-old infant with an intensely pruritic rash was noted to scratch his skin constantly.

An 11-month-old Malay boy presented with a 6-month history of an intensively pruritic scaly rash characterized by crusting and excoriation over the body (Figures 1 and 2). The infant was seen at 8 months of age by his family physician. He was misdiagnosed to have atopic dermatitis with secondary bacterial infection at 10 months of age, and he was treated with topical mometasone furoate cream daily for two weeks, oral cloxacillin for 7 days, and an emollient several times a day. In spite of the treatment, there was no improvement of the eruption and no relief of the itch. A serum immunoglobulin E (IgE) was performed and was found to be normal. Because of the intense pruritus and the lack of improvement with the current therapy, the infant was referred to one of us (KFL) at 11 months of age.

Past medical history revealed that the infant was born to a 26-year-old primigravida woman at 39 weeks gestation following an uncomplicated pregnancy and delivery. He was exclusively breastfed for 6 months, at which time solid food was introduced. The developmental milestones were normal. His past medical history was otherwise unremarkable, and he had not been on any medications until 8 months of age which was 3 months after onset of the rash.

Family history revealed that both parents had an intensely pruritic erythematous papular eruption affecting the interdigital web spaces and lateral aspects of fingers approximately 2 to 3 months after the onset of the eruption in the infant. The parents did not have any crusted lesions. They were seen by a dermatologist, who made the diagnosis of scabies and treated with 5% permethrin cream with reduction of the pruritus and improvement of the lesions. On further questioning, the babysitter was found to have crusted scabies. She was seen and treated by a dermatologist.

**Figure 3:**
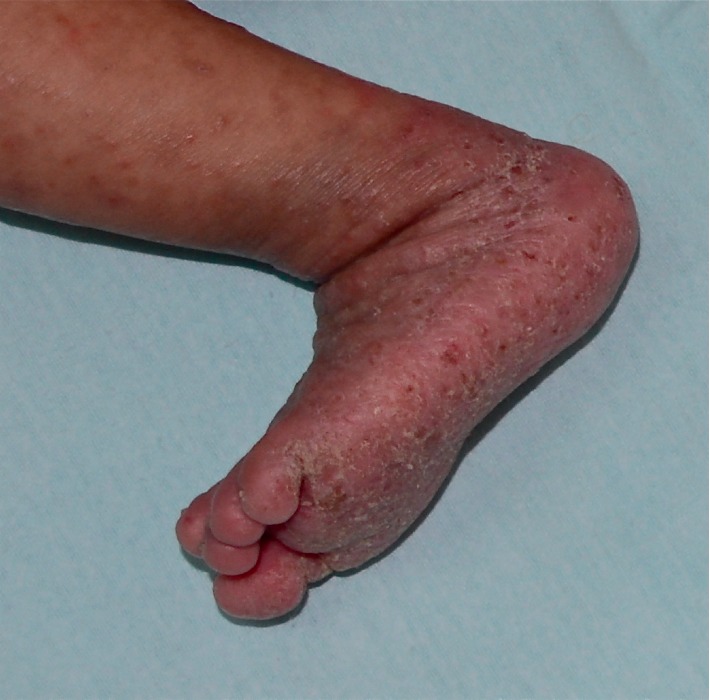
Diffuse, crusted, hyperkeratotic, and erythematous patches and plaques, involving the left leg and foot.

On examination, the infant was well nourished and not in distress. His weight was 8.8 kg, height 74 cm, temperature 37°C, heart rate 78 beats per minute, and respiratory rate 28 breaths per minute. Diffuse, scaly, crusted, hyperkeratotic, erythematous patches and plaques were seen over the body. Some of the lesions were excoriated. The lesions were accentuated on the groins, palms, and soles (Figure 3). The rest of the physical examination was normal.

**Figure 4:**
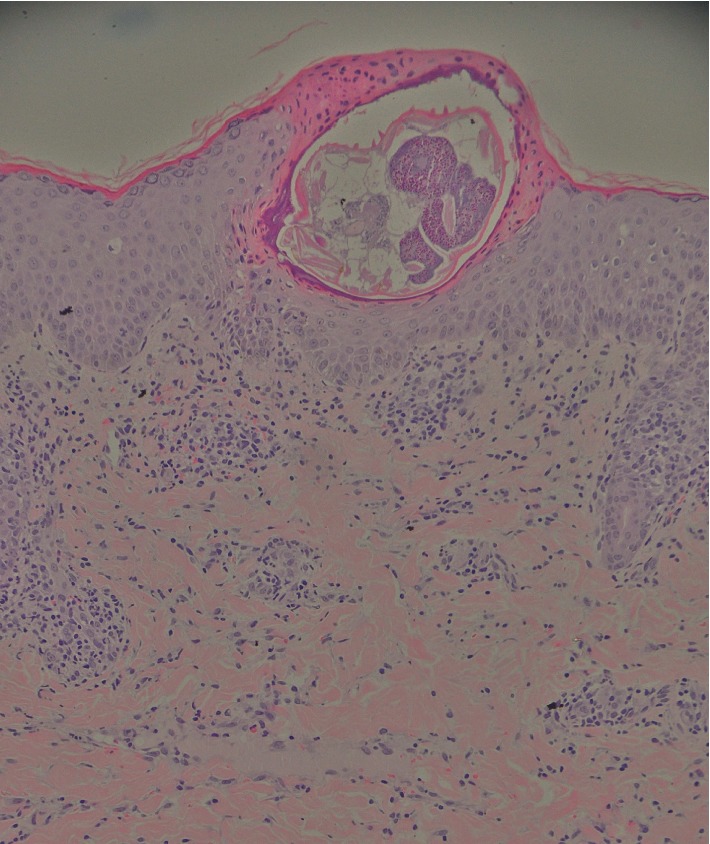
Histological examination of a skin biopsy specimen showed acanthosis, parakeratosis, spongiosis, and a scabies mite within the epidermis. It also showed superficial perivascular and diffuse infiltrate of lymphocytes and histiocytes within the dermis (Hematoxylin-eosin stain, original magnification ×200).

Direct microscopic examination of skin scrapings revealed numerous scabies mites and eggs. A skin biopsy was performed on one of the lesions which revealed the scabies mite within the epidermis (Figure 4). A diagnosis of crusted scabies was made. His complete blood cell count, differential count, T-cell and B-cell subsets, quantitative immunoglobulins, and HIV test were all normal.

**Figure 5:**
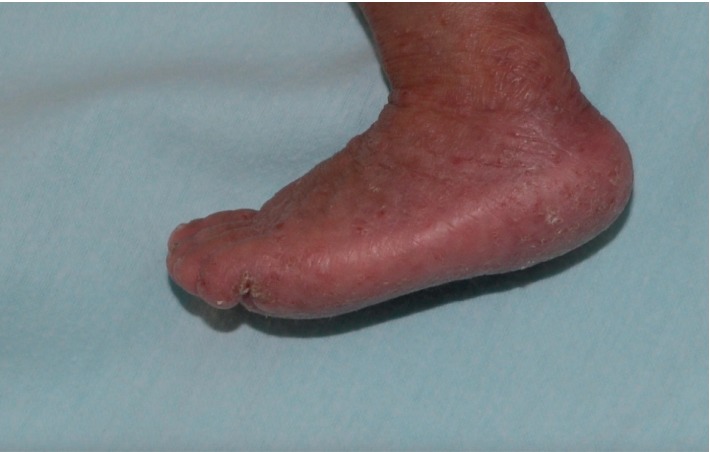
Crusted scabies lesions on the left leg and left foot 3 weeks after treatment.

**Figure 6:**
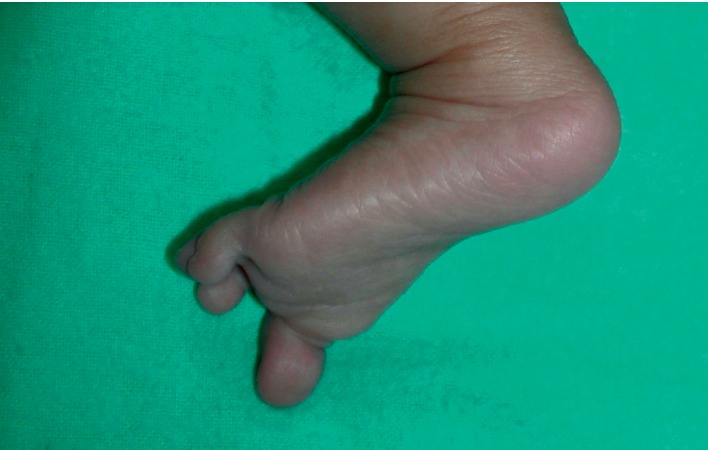
Complete resolution of crusted scabies lesions on the left leg and left foot 6 weeks after treatment.

The infant was treated with overnight application of topical 5% permethrin cream to the entire body weekly for a total of 6 weeks. There was complete resolution of cutaneous lesions at the end of the treatment (Figures 5 and 6).

## 3. Discussion

Crusted scabies is seldom reported in infancy, especially in healthy infants. The condition was originally described in Norway by Danielssen and Boeck as a type of scabies infestation caused by millions of mites in patients with leprosy [7]. Crusted scabies is characterized by widespread erythroderma, hyperkeratosis, and crusting of the skin [8]. Lesions tend to be exaggerated on the soles, palms, ears, and extensor surface of the elbows [8, 9]. The crusts may be scaly and loose or adherent and thick [9]. On removal of the crust, the skin surface appears smooth, velvety, and red [8]. Crusted scabies usually occurs in immunocompromised individuals such as those with congenital immunodeficiency disorders (especially cell-mediated immunodeficiency), acquired immunodeficiency syndrome (AIDS)/human immunodeficiency virus (HIV) infection, immunosuppressive treatment (e.g., corticosteroids, calcineurin inhibitors, and cytotoxic drugs), graft-versus-host disease, malignancies (e.g., leukemia and lymphoma), and systemic diseases (diabetes mellitus, systemic lupus erythematosus, dermatomyositis, chronic mucocutaneous candidiasis, and dystrophic epidermolysis bullosa) [6, 8, 10, 11]. Other predisposing factors include malnutrition, physical debilitation (senility, sensory or motor neuropathy, leprosy, and paraplegia), and mental retardation (especially Down syndrome) [2, 5, 8, 12, 13].

Our patient is unique in that he was healthy and not immunocompromised. He had scaly, crusted, and excoriated lesions characteristic of crusted scabies 5 months before he was treated with topical corticosteroids. A perusal of the literature revealed only one case occurring in infancy [12]. In 2004, Baysal et al. reported a 4½-month-old otherwise healthy girl with crusted scabies who presented with a 4-month history of pruritic erythematous, scaly, and excoriated papules on the trunk and hyperkeratotic crusted papules on the palms and soles. The diagnosis was confirmed by observation of a mite on microscopic examination of material scraped from the lesions. There were a few case reports of immunocompromised infants with crusted scabies [1, 10, 14, 15]. In 2006, Ruiz-Maldonado reported a 2½-month-old infant with scabies who was misdiagnosed as atopic dermatitis at 3 weeks of age and treated with topical pimecrolimus 1% cream twice a day [15]. A total of 120 gm was applied over a 3-week period, with improvement of the pruritus and appearance of widespread papules, nodules, and erythema on his trunk and limbs. Crusts and burrows were noted on the feet and hands. Scrapings of crusts, papules, and nodules yielded 5 to 12 mites each amid abundant eggs, larvae, and fecal pellets. In 2009, Gualdi et al. reported a 3-month-old girl with atopic dermatitis since birth [1]. The patient was treated with topical betamethasone and gentamicin. Oral betamethasone in a dose of 0.5 mg/day was later added to the treatment with worsening of the dermatitis and emergence of monomorphic vesicles and papules. Microscopic examination of scraped scales from some lesions showed numerous scabies mites. In 2014, Rose et al. reported crusted scabies in a 4-month-old human immunodeficiency virus- (HIV-) positive infant who presented with papular lesions all over the body and crusted scaly lesions over the soles and palms [14]. In 2017, Lima et al. described a 4-month-old Mulatto boy who had miliaria rubra-like lesions on the neck and folds of the upper and lower limbs since 2 months of age [10]. The infant was treated with various medications including oral prednisolone (0.8 mg/kg/day) for 7 days and a single-dose ampoule of betamethasone given intramuscularly. Since then, the lesions became crusted and spread to the trunk, scalp, and face. The diagnosis of crusted scabies was made based on typical dermoscopic and histopathologic findings.

Crusted scabies is a highly contagious infestation because of the high burden of mites in the epidermis. Early diagnosis is therefore important. Typically, pruritus is minimal or absent in crusted scabies [2, 14]. In the present case, a misdiagnosis of atopic dermatitis was made which might have been ascribed to the intensely pruritic nature of the lesions and unawareness or unfamiliarity of crusted scabies in healthy infants. Atopic dermatitis is a chronic relapsing dermatosis characterized by pruritus, erythema, vesiculation, exudation, excoriation, crusting, scaling, xerosis, and sometimes lichenification [16, 17]. In infancy, the eruption often affects the face and scalp although the extensor surfaces of the extremities and the trunk may also be affected [16, 17]. The term “milk scurf” or “milk crust” refers to the occurrence of yellowish crusts on the scalp which resemble scalded milk [17]. The nose is often spared and is referred to as the “head light” sign [17]. This case serves to remind readers to always consider crusted scabies in the differential diagnosis of atopic dermatitis if the lesions are crusted and hyperkeratotic regardless of whether the lesions are pruritic, especially if the lesions fail to respond to steroid treatment. Other differential diagnoses include seborrheic dermatitis, drug eruption, insect bites, contact dermatitis, ichthyosis vulgaris, Langerhans cell histiocytosis, cutaneous lymphoma, psoriasis, Darier disease, palmoplantar keratoderma, dermatitis herpetiformis, and lichen planus [8, 12, 14]. The distinctive features of the aforementioned usually allow a straight forward differentiation from crusted scabies. Demonstration of the scabies mites, eggs, or fecal pellets (scybala) in skin scraping or skin biopsy differentiates crusted scabies from other conditions. Physicians should be vigilant on the occurrence of crusted scabies in infants, whether they are immunocompromised or immunocompetent, as delay in diagnosis and treatment may lead to dissemination of the infestation.

## 4. Conclusion

Crusted scabies is extremely contagious and usually occurs in immunocompromised individuals. The condition has rarely been reported in healthy infants. We report an 11-month-old healthy infant with pruritic crusted scabies which started at the age of 5 months. The occurrence of crusted scabies in healthy infants may be more common than is generally appreciated. This case report highlights the need to consider crusted scabies in healthy infants with crusted, hyperkeratotic lesions so that an early diagnosis can be made and treatment initiated.
